# Mitochondrial dysfunction is an important cause of neurological deficits in an inflammatory model of multiple sclerosis

**DOI:** 10.1038/srep33249

**Published:** 2016-09-14

**Authors:** Mona Sadeghian, Vincenzo Mastrolia, Ali Rezaei Haddad, Angelina Mosley, Gizem Mullali, Dimitra Schiza, Marija Sajic, Iain Hargreaves, Simon Heales, Michael R. Duchen, Kenneth J. Smith

**Affiliations:** 1 Department of Neuroinflammation, Queen Square Multiple Sclerosis Centre, UCL Institute of Neurology, WC 1N 1PJ, London, UK; 2 Neurometabolic Unit, National Hospital for Neurology and Neurosurgery, London, UK; 3 Chemical Pathology, Great Ormond Street Children’s Hospital, London, UK; 4 Cell and Developmental Biology, University College London, Gower Street, WC1E 6BT, London, UK

## Abstract

Neuroinflammation can cause major neurological dysfunction, without demyelination, in both multiple sclerosis (MS) and a mouse model of the disease (experimental autoimmune encephalomyelitis; EAE), but the mechanisms remain obscure. Confocal *in vivo* imaging of the mouse EAE spinal cord reveals that impaired neurological function correlates with the depolarisation of both the axonal mitochondria and the axons themselves. Indeed, the depolarisation parallels the expression of neurological deficit at the onset of disease, and during relapse, improving during remission in conjunction with the deficit. Mitochondrial dysfunction, fragmentation and impaired trafficking were most severe in regions of extravasated perivascular inflammatory cells. The dysfunction at disease onset was accompanied by increased expression of the rate-limiting glycolytic enzyme phosphofructokinase-2 in activated astrocytes, and by selective reduction in spinal mitochondrial complex I activity. The metabolic changes preceded any demyelination or axonal degeneration. We conclude that mitochondrial dysfunction is a major cause of reversible neurological deficits in neuroinflammatory disease, such as MS.

*In vivo* imaging has been used to examine how mitochondrial damage can contribute to axonal degeneration in experimental autoimmune encephalomyelitis (EAE), a neuroinflammatory disease often used as a model of MS^1^, but the relationship between mitochondrial (dys)function and the expression of neurological deficits such as paralysis has not been examined. We have assessed the relationship between changes in axonal and glial mitochondrial function and trafficking and the expression of neurological deficits over the course of EAE, using confocal *in vivo* imaging of the spinal cord in mice, emphasising events at the onset of disease expression. Neurological deficits in inflammatory demyelinating disease have been widely attributed to impaired axonal conduction arising from demyelination^2^. However, increasing clinical, imaging and biopsy evidence show that inflammation also plays a major role^3^,^4^, Indeed, a biopsy study in patients with MS concluded that: “Inflammation alone may be sufficient to cause significant clinical deficits without demyelination^5^. However, the mechanism by which inflammation causes loss of function remains unresolved, although roles for cytokines^6^, reactive oxygen species and nitric oxide^7^,^8^,^9^ have been proposed. Some of these factors can impair mitochondrial function^10^, and increasing evidence points to an energy deficit as a major feature of the brain and spinal cord in multiple sclerosis (MS) e.g. refs 11, 12, 13, 14, 15 implicating an energy deficit in the pathophysiology of the functional deficit.

Our findings reveal profound changes in mitochondrial function that parallel the equally profound changes in neurological function. Loss of mitochondrial function at the start of disease expression was accompanied by the increased expression of a key astrocytic glycolytic enzyme.

These data place mitochondrial dysfunction at the centre of the pathophysiology of demyelinating disease of the CNS.

## Results

### Animal numbers at different stages of EAE are added

Mice with EAE expressed no neurological deficits for the first 9–11 days post immunization (p.i.), but then progressively developed neurological deficits, which reached a peak at ~18 days. Animals developed weakness and then paralysis of the tail, escalating to include weakness and paralysis of the hindlimbs. The mice then entered a period of remission, which, in 9 out of 14 mice, fully restored function by day 22 p.i. A period of relapse ensued from approximately day 26 during which the same deficits were re-expressed and sometimes became more severe over the remainder of the study period (47 days p.i.).

### Mitochondrial dysfunction parallels neurological deficits

**Figure 1:**
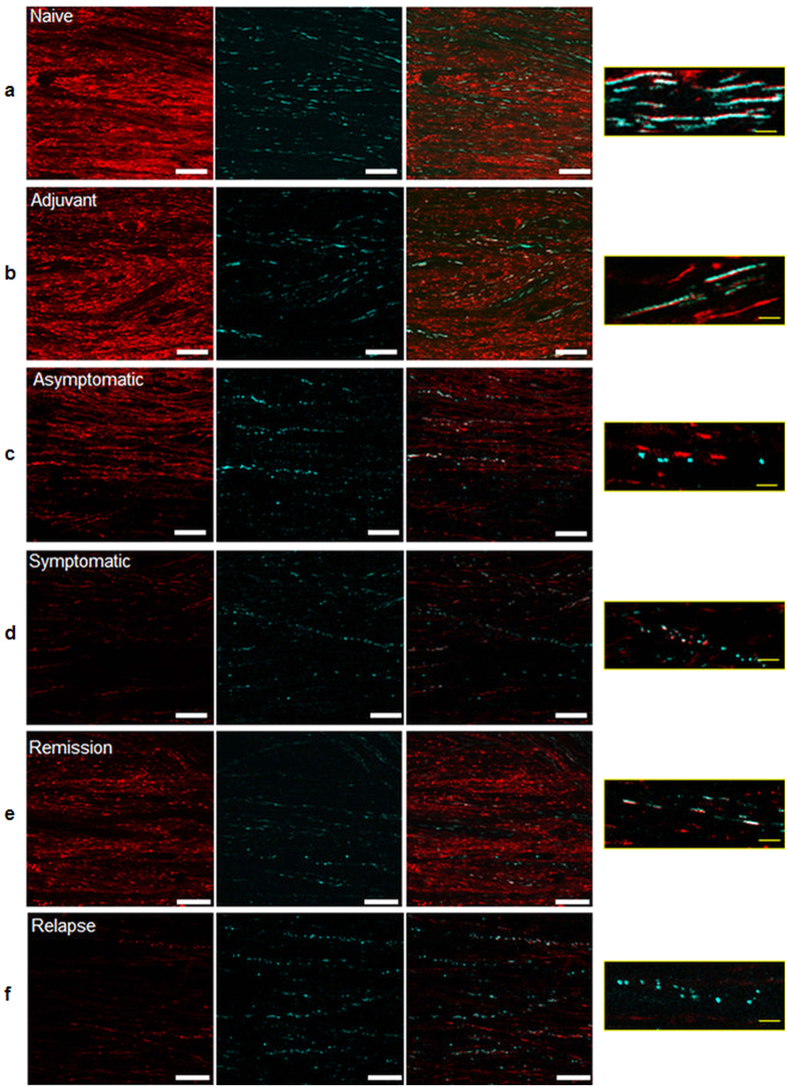
Axonal mitochondrial function varies in parallel with the expression of neurological deficits. (**a**,**b**) Confocal images of naïve and adjuvant control mouse spinal cords showing labelling for TMRM (red) and axonal CFP^+^ mitochondria. The bright TMRM fluorescence co-localises with the CFP fluorescence (see images at higher magnification) demonstrating that the adjuvant control and naive axonal mitochondria often have long and thin morphology and bright TMRM fluorescence indicating that they are polarised. (**c**) TMRM fluorescence intensity is decreased in the spinal cord of asymptomatic animals shortly before the expected onset of neurological deficits, and higher magnification shows fragmented CFP^+^ axonal mitochondria with very low, or absent, TMRM accumulation. (**d**) Significant reduction in TMRM fluorescence intensity and mitochondrial length at the onset of neurological deficits. (**e**) Increased TMRM fluorescence intensity in the spinal cord of a mouse in remission. (**f**) TMRM fluorescence intensity is very low in the spinal cord of mice in relapse, and the mitochondria are very fragmented. Scale bar = 10 μm.

**Figure 2:**
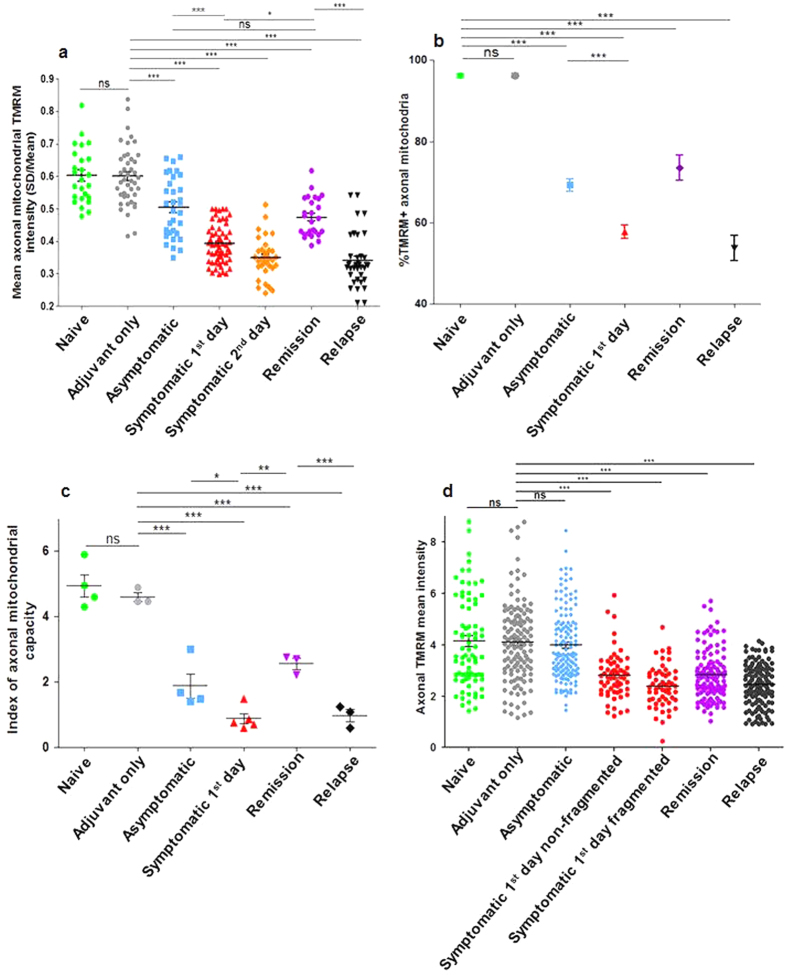
(**a**) Mean TMRM intensity in CFP^+^ axonal mitochondria in different groups of animals expressed as the coefficient of variance of TMRM pixel intensities of mitochondria against axonal TMRM intensities (standard deviation of the mean intensity divided by the mean intensity). TMRM intensity was significantly lower in animals with EAE at all stages of disease. The onset of disease expression was marked by very severe mitochondrial depolarisation, but during remission, the membrane potential improved to the level observed in asymptomatic mice. During relapse, the mean TMRM intensity was particularly reduced. (**b**) Percentage of CFP^+^ axonal mitochondria expressing any detectable TMRM fluorescence. Virtually all axonal mitochondria were TMRM^+^ in naïve and adjuvant control mice (perhaps 100% were TMRM^+^ allowing for some mitochondria to move between scans for CFP and TMRM). Significantly fewer axonal mitochondria were positive for any level of TMRM fluorescence in animals at all stages of EAE. There were significantly fewer TMRM^+^ mitochondria at the onset of neurological deficits, but during remission the percentage of TMRM^+^ mitochondria increased to the level observed in asymptomatic mice. During subsequent relapse, the percentage of axonal mitochondria with any detectable labelling for TMRM was the lowest observed at any stage. Data are presented as mean ± S.E.M. (**c**) Index of axonal mitochondrial capacity per mouse in each group. The changes in this index mirrored the changes described in (**a**,**b**), such that mitochondrial dysfunction paralleled the expression of neurological deficits. (**d**) Axonal TMRM fluorescence. No depolarisation of axons was detected in naïve, adjuvant control or asymptomatic animals, but axons were significantly depolarised at the onset of neurological deficits. Axons with fragmented mitochondria were more depolarized than those in which fragmentation was less prominent. During remission, the mean axonal TMRM intensity improved, and more axons showed brightness within the control range. During relapse, the axonal TMRM intensity decreased to the levels observed in axons with fragmented mitochondria on the first day of neurological deficits.

The spinal cord was exposed by removal of the T13-L2 lamina and mitochondrial membrane potential, a measure of mitochondrial function, was revealed using the cationic, potentiometric dye, tetramethylrhodamine methyl ester (TMRM, Invitrogen, UK). In naïve and adjuvant-only control mice, all the mitochondria, including all CFP labelled mitochondria (i.e. by definition, axonal mitochondria that are transgenic for expression of cyan fluorescent protein (CFP) in axonal mitochondria driven by a neuronal promoter – see Methods), were functional throughout the spinal cord, with bright TMRM fluorescence reporting a high membrane potential (Figs 1a and 2a,b). In contrast, in animals with EAE, many mitochondria were depolarised (Figs 1d and 2a,b) – i.e. they showed CFP fluorescence but weak or minimal TMRM fluorescence. The dysfunction started before the onset of neurological deficit, indicated by a significant loss of TMRM fluorescence in asymptomatic mice with EAE when other mice immunised on the same day had already started to express hind limb and tail weakness (Fig. 1c). In these animals, the mean fluorescence intensity of TMRM in polarised axonal mitochondria was decreased by 19% compared with control mice treated only with adjuvant (p < 0.001) (Fig. 2a), and 36% of small axonal mitochondria (1.5–2.5 μm in length) were completely or almost completely depolarised (showing CFP but no detectable TMRM fluorescence), comprising 28% of the total axonal mitochondrial mass (Supplementary Fig. 1a). The index of axonal mitochondrial capacity, a measure of the mass of polarised mitochondria, also dropped significantly before the appearance of neurological signs, by 57% and 62% compared with adjuvant treated (p < 0.001) and naïve mice (p < 0.001) respectively (Fig. 2c).

The onset of neurological deficits was associated with an additional and significant deterioration of mitochondrial function compared with adjuvant only and asymptomatic control mice (Figs 1D and 2a,b). TMRM fluorescence intensity in partially polarised mitochondria was decreased by 39% and 43% on the first and second days of neurological signs respectively, compared with control mice treated only with adjuvant (Fig. 2a). In addition, the proportion of axonal mitochondria that were totally depolarised (TMRM^−^) increased to 54% of the small mitochondria (1.5–2.5 μm in length), comprising 40% of the total axonal mitochondrial mass (p < 0.001) compared with control mice treated only with adjuvant (Figs 1d and 2b and supplementary Fig. 1b). At this stage the index of axonal mitochondrial capacity decreased significantly by 81% and 82% compared with naïve mice (p < 0.001), and control mice treated only with adjuvant (p < 0.001) respectively (Fig. 2c).

During remission, with complete clinical recovery of neurological function, axonal mitochondrial function recovered to the level observed in asymptomatic mice, with no significant difference between the groups (Figs 1e and 2(a,b)). The TMRM fluorescence in partially polarised mitochondria was only decreased by 26% compared with control mice treated only with adjuvant (p < 0.01) (Fig. 2a), and only 19% of the total mass of axonal mitochondria were entirely depolarised, representing a significant improvement over animals on the first day of neurological deficit (p < 0.01) (Fig. 2b and supplementary Fig. 1c). During remission, the index of axonal mitochondrial capacity recovered significantly (~34% improvement) compared with mice examined on the first day of deficits, or compared with mice in relapse (Fig. 2c). The mitochondrial depolarisation returned concurrently with the relapse of neurological signs. Thus in animals with chronic EAE examined 6–7 weeks post immunization, TMRM fluorescence was again decreased by 41% compared with control mice treated only with adjuvant (p < 0.001) (Figs 1f and 2(a,b)), and approximately half the axonal mitochondria were totally depolarized (absence of TMRM signal) (p < 0.001 compared with control mice treated only with adjuvant) (Fig. 2b, and supplementary Fig. 1d). In these mice the index of axonal mitochondrial capacity decreased significantly by 78% compared with adjuvant only treated mice, and also significantly compared with mice in remission (p < 0.001) (Fig. 2c).

In summary, neurological deficits appear when the axonal mitochondrial capacity index is approximately 20% of normal. During remission, the capacity index partially recovered to 52% of normal. The mitochondrial capacity index thus changes in parallel with the changes in neurological deficits.

### Mitochondrial respiratory chain complex I activity is decreased on the first day of neurological signs

The enzyme activity of mitochondrial respiratory chain complex I was significantly reduced at the onset of disease compared with control animals immunised with adjuvant only, but the activity of complexes II-III and IV was unchanged (Supplementary Fig. 2a–c).

### Axonal depolarisation accompanies the expression of neurological deficits

The onset of neurological deficits was marked by the depolarisation of CNS *axons*. Thus the axons in asymptomatic animals were not depolarised, despite the presence of a degree of mitochondrial depolarisation (Fig. 2d), but axons with many dim and fragmented mitochondria were more depolarised than those in which these abnormalities were less prominent (Fig. 2d). Remission was associated with an increase in the number of axons with TMRM fluorescence around the normal mean, although interestingly the mean axonal TMRM fluorescence was not significantly different from that in symptomatic animals with non-fragmented mitochondria. During relapse the axons were as depolarised as during the first day of neurological deficit (Fig. 2d).

### Mitochondrial dysfunction is associated with infiltrating immune cells

Inflammatory cells are readily identified within inflamed spinal cords and in the day(s) prior to the onset of disability (i.e. in asymptomatic animals with EAE where some time-matched cohort animals already exhibit a deficit) as sporadic clusters of perivascular infiltrating immune cells. Most of the perivascular inflammatory cells at the onset of neurological deficits were identified as macrophages on morphological criteria, an interpretation supported by fluorescence labelling with the fluorescent NO reporter DAF-FM, indicating the production of nitric oxide (Supplementary video 1). Subsequent immunohistochemical analysis confirmed that the majority of the recruited perivascular cells were CD45^+^ macrophages, of which the majority were positive for iNOS immunofluorescence (Supplementary Fig. 3a). CD3^+^ T cells were also present, in smaller numbers, particularly near the pial surface of the dorsal columns. Nitric oxide production signalled by DAF-FM was not detected by confocal imaging in any cell types other than putative macrophages. Nitric oxide reacts with superoxide to form peroxynitrite, which reacts with tyrosine residues to form 3-nitrotyrosine (3-NT). Immunohistochemical labelling for 3-NT was strongly positive in the spinal cord white and grey matter on the first day of neurological signs, primarily co-localised with CFP^+^ axonal mitochondria (Supplementary Fig. 3b, white arrow), but was absent in naïve or adjuvant-only control mice (Supplementary Fig. 3a). During remission, when mitochondrial function improved (see above), nitric oxide production was undetectable, very few CD45^+^ macrophages cells were seen on histology, and 3-NT was no longer detected. During relapse, there was no labelling *in vivo* with DAF-FM, or labelling for iNOS expression upon immunohistochemistry, but 3-NT immunoreactivity was widespread, including co-localisation with CFP^+^ mitochondria.

Microglial cells did not express MHC-II (i.e. OX-6) in asymptomatic animals, or at the onset of the disease, despite the presence of mitochondrial damage. Labelling for Iba1 revealed no increase in the number of microglia in asymptomatic mice, but the number was increased on the first day of neurological deficits.

**Figure 3:**
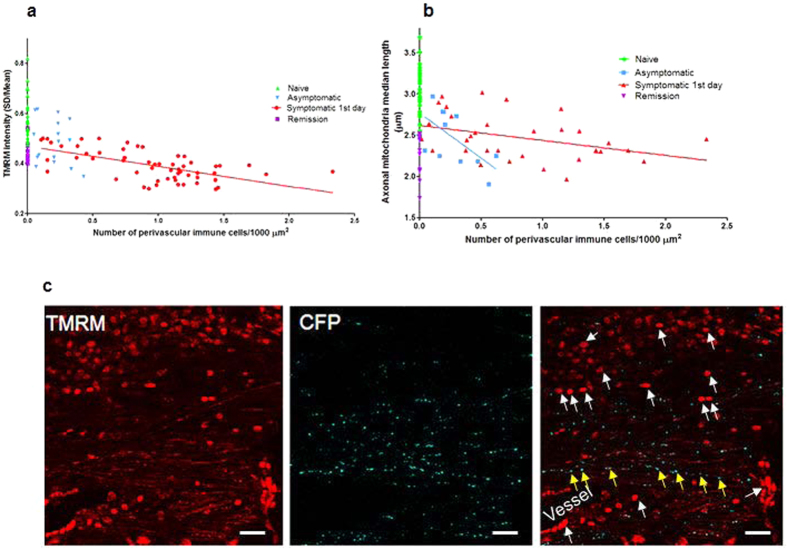
Mitochondrial fragmentation and decreased TMRM intensity correlates with the magnitude of inflammation on the first day of neurological deficits. (**a**) Correlation analysis of symptomatic animals on the first day of neurological deficits shows that TMRM intensity is lower in areas with more infiltrating immune cells, p < 0.001 and r^2^ = 0.48. In naïve, asymptomatic and remitting mice there was no significant correlation, p > 0.5 and r^2^ = 0.06. (**b**) Correlation analysis for the medial length of mitochondria reveals that mitochondrial size is inversely correlated with the number of infiltrating immune cells on the first day of neurological deficits p < 0.001 and r^2^ = 0.30, with a mild similar but not significant correlation in asymptomatic mice, although the number of immune cells in asymptomatic mice is very low, p > 0.05, r^2^ = 0.37. (**c**) Confocal images showing the spinal cord of a mouse on the first day of neurological deficits. Infiltrating immune cells (white arrows) associated with blood vessels and the perivascular space are labelled with TMRM, but most of the CFP^+^ axonal mitochondria are fragmented and depolarised (TMRM^−^) (yellow arrows). (Scale bar 20 μm).

In asymptomatic animals, there was no obvious spatial correlation between infiltrating cells and abnormal mitochondria. In contrast, with the onset of neurological deficits, there was not only a large increase in the number of infiltrating cells, but now the inflammatory cells and dysfunctional mitochondria were clearly spatially correlated. The number of infiltrating inflammatory cells was significantly correlated with both the loss of mitochondrial function (p < 0.001) and the increased mitochondrial fragmentation (p < 0.02) (Fig. 3a–c).

### Mitochondrial dysfunction is associated with mitochondrial fragmentation

**Figure 4:**
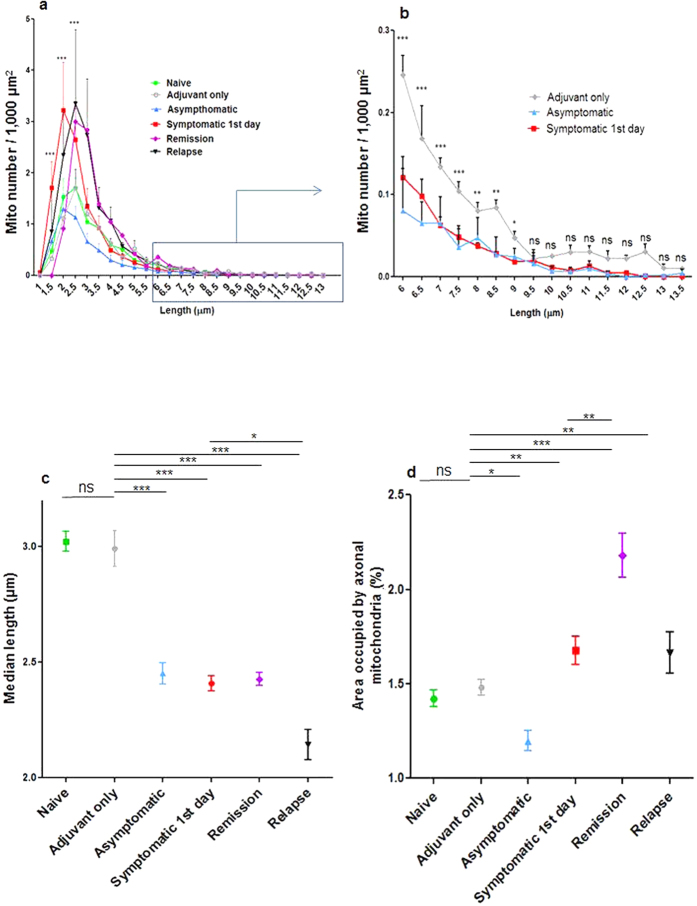
Mitochondrial fragmentation and increase in mass on the first day of neurological deficits. (**a**) Graph showing the frequency distribution of CFP^+^ axonal mitochondria of different lengths. In naïve mice the mitochondrial length ranges between 1.5–13 μm, with the majority between 1.5–6 μm, but in mice with a neurological deficit the number of short axonal mitochondria was doubled in symptomatic animals (increase of 104% in mitochondria 1.5–2.5 μm in length) and there was a significant decrease in the number of longer axonal mitochondria (6–9 μm in length), compared with asymptomatic and adjuvant only mice (graph **b**, which shows part of graph **a** in greater detail). The increase in the number of short mitochondria persisted during remission and subsequent relapse, compared with adjuvant only mice. (**c**) The median length of axonal mitochondria was significantly decreased at all stages of EAE, including in asymptomatic mice, and especially during relapse. (**d**) Mitochondrial mass (measured by the area of axoplasm occupied by CFP^+^ mitochondria) decreased significantly in asymptomatic mice, but it increased significantly on the first day of neurological deficits, even when compared with adjuvant only animals (although the mass increased, many of the mitochondria were not functional). During remission, the axonal mitochondrial mass increased even further, but reduced during relapse to the level exhibited at the onset of neurological deficits. Comparisons were made by two-way ANOVA using GraphPad Prism software (GraphPad, San Diego, CA) accompanied with Bonferroni post-hoc analysis. All comparisons were performed within and between groups. *p < 0.05, **p < 0.01, ***p < 0.001.

Axonal mitochondria are normally long and thin (Fig. 1a, Box), ranging between 1.5–13 μm in length, with the majority between 1.5–6 μm (Fig. 4a). However in animals with EAE the mitochondria were much shorter (p < 0.001; Fig. 4c), and their number was doubled (p < 0.001) (Fig. 4a, 1c box), compared with control mice treated only with adjuvant, even before the onset of neurological signs. There was a corresponding and significant decrease in the number of longer axonal mitochondria (6–9 μm) compared with control mice treated only with adjuvant, both before the onset of neurological signs, and on the first day of expression of deficits (Fig. 4b). Mitochondrial length did not recover when animals went into remission but the shortening became even more exaggerated during relapse (6–7 weeks p.i.) (Fig. 1f box) compared with adjuvant controls or with mice on the first day of neurological deficits (p < 0.001 and p < 0.05, respectively).

### Mitochondrial mass increases at the onset of symptoms

Axonal mitochondrial abundance changed significantly during the course of EAE (Fig. 4d). Mitochondrial mass in axons decreased significantly in the prodromal phase, whether measured by the integrated length of all mitochondria in a given portion of axon, or by the area of CFP^+^ fluorescence (p < 0.05; compared with naïve and adjuvant-treated mice) (Fig. 4d). In contrast, mitochondrial mass (measured by integrated length or area) increased significantly compared with adjuvant controls at the onset of neurological signs (p < 0.01) (Fig. 4a,d), and increased still further during remission compared either with control mice treated only with adjuvant (p < 0.001) or with symptomatic animals on the first day of neurological deficit (p < 0.01) (Fig. 4d). During relapse (6–7 weeks post immunisation) the axonal mitochondrial mass decreased to levels equivalent to symptomatic animals on the first day of deficit, still remained significantly higher than in adjuvant controls (p < 0.01) (Fig. 4d). Note that although mitochondrial mass increased at the onset of disease, most of the mitochondria were depolarised and therefore were not functional.

### Mitochondrial trafficking is reduced at the onset of neurological deficits

**Figure 5:**
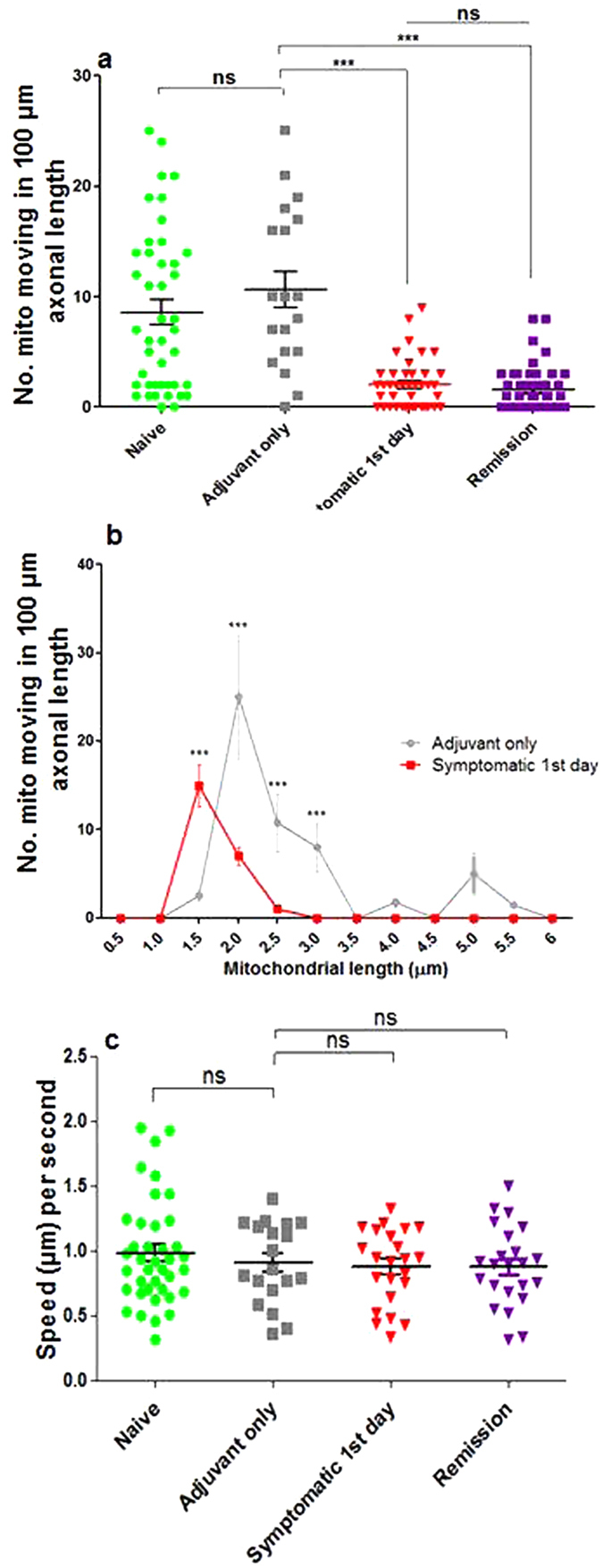
Reduced number of motile axonal mitochondria in dorsal column axons on the first day of neurological deficits. (**a**) The number of moving CFP^+^ axonal mitochondria expressed as the number moving per 100 μm of axonal length was significantly reduced on the first day of neurological signs compared with adjuvant only control mice: the movement did not recover during remission. (**b**) On the first day of neurological deficits the large majority of motile axonal mitochondria were 1.5–2 μm in length, whereas in adjuvant only animals the motile axonal mitochondria were mostly 2–3 μm in length (p < 0.001). (**c**) The speed of the motile axonal mitochondria was not significantly different between mice at different stages of EAE. Comparisons were made by two-way ANOVA using GraphPad Prism software (GraphPad, San Diego, CA) accompanied with Bonferroni post-hoc analysis. All comparisons were performed within and between groups. *p < 0.05, **p < 0.01, ***p < 0.001, ns = p > 0.5.

Mitochondrial movement was significantly decreased at the onset of neurological deficits, such that the mean number of motile axonal mitochondria in the dorsal column axons was 8.58 ± 1.11 and 10.63 ± 1.66 per 100 μm axonal length in naïve and adjuvant control mice respectively, but this was reduced to only 2.04 ± 0.34 per 100 μm axonal length on the first day of neurological deficits in EAE (p < 0.001 compared with adjuvant control mice) (Fig. 5a,b and supplementary video 2). Mitochondrial trafficking did not recover during remission compared with the first day of neurological dysfunction (1.62 ± 0.30 vs. 2.05 ± 0.34 per 100 μm axonal length, p = 0.596) (Fig. 5a). The direction of mitochondrial movement was relatively balanced in control dorsal column axons (56% moving rostrally, vs. 44% moving caudally, (Supplementary Fig. 4b) and neither this balance, nor the speed (Fig. 5c), changed significantly during EAE.

In all the mice studied, only short polarised mitochondria (i.e. TMRM^+^) were motile. All depolarised (i.e. CFP^+^, TMRM^−^) mitochondria, together with a sub-population of the polarised mitochondria, were stationary (Supplementary Fig. 4a, movie snap shots). On the first day of neurological deficits, the large majority of motile axonal mitochondria were very short with six times more mitochondria shorter than 1.5 μm in length compared with adjuvant only mice (p < 0.001) (Fig. 5b).

### Absence of demyelination on the first day of neurological deficits

Histological examination of the spinal cord in transverse and longitudinal sections failed to reveal any evidence of demyelination or axonal damage on the first day of neurological deficits compared with naïve and adjuvant-only control animals, whether viewed in luxol fast blue (LFB) stained longitudinal sections, or at high resolution in semi-thin resin sections (700 nm thick; Supplementary Fig. 5a–e), including the animals that had been imaged confocally. Axonal damage (indicated by SMI32 labelling) was only sparsely present throughout the dorsal and ventral columns when examined immunohistochemically in frozen sections on the first day neurological deficits. Such damage was absent in naïve animals, adjuvant controls and asymptomatic mice with EAE. Longitudinal sections through the SMI32^+^ axons on the first day of neurological deficit revealed many short mitochondria (Supplementary Fig. 5f), in contrast to the long and thin mitochondria present in SMI32^−^ axons.

### Disruption of astrocytes in the inflamed areas with increased glycolytic capacity in activated astrocytes at the onset of neurological deficits

**Figure 6:**
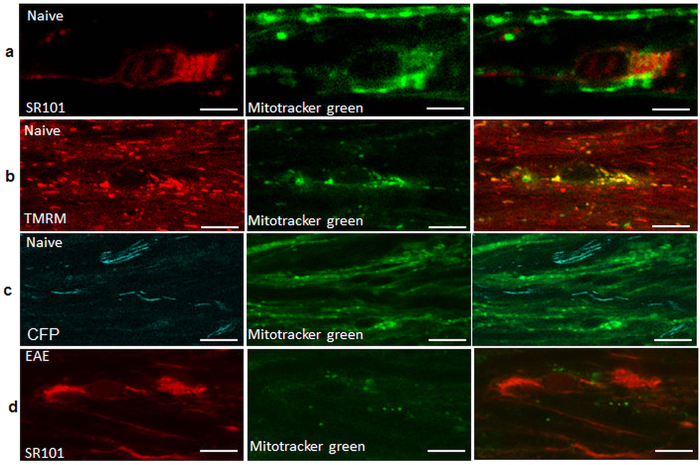
Dysfunction of astrocytes on the first day of neurological signs. (**a**) *In vivo* co-localisation of Mitotracker green with the astrocyte-specific marker SR101, and (**b**) with TMRM, showing polarisation of mitochondrial astrocytes in naïve mice. (**c**) Labelling with Mitotracker green does not colocalize with CFP^+^ axonal mitochondria in naïve mice. (**d)** Co-labelling of Mitotracker green and SR101 is absent on the first day of neurological deficits, showing depolarisation of astrocytic mitochondria at this time.

**Figure 7:**
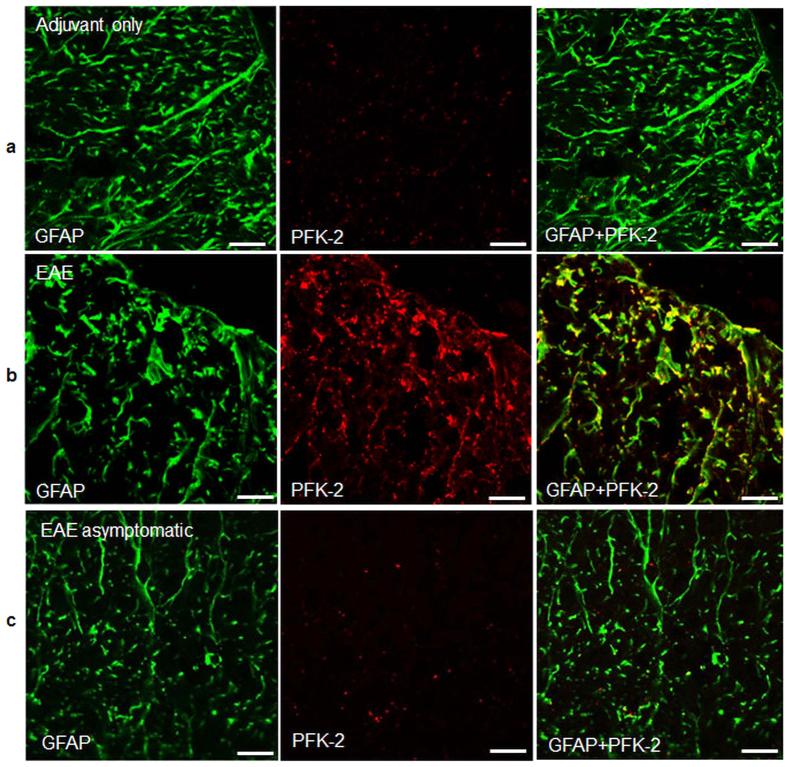
Increase in glycolytic capacity in activated astrocytes on the first day of neurological deficits. (**a**) Immunohistochemical labelling for the astrocytic marker GFAP (green) and for the glycolytic marker enzyme PFK-2 (red) in adjuvant only mice. (**b**) In animals with EAE on the first day of neurological deficits the astrocytes lose their normal long and thin morphology and gain labelling for PFK-2. (**c**) No PFK-2 labelling was observed in the astrocytes of asymptomatic mice.

The mitochondrial dye Mitotracker Green preferentially labelled mitochondria in glial cells (it did not cross myelin to label axonal mitochondria) including cells marked by the astrocyte-specific marker sulforhodamine 101 (SR101), establishing the presence of functional mitochondria in astrocytes (Fig. 6a,b). In EAE, virtually no Mitotracker Green-labelled mitochondria could be identified in inflamed areas (Fig. 6c), revealing that astrocytic mitochondria as well as axonal mitochondria were depolarised. Subsequent immunohistochemical examination revealed that the perivascular inflammatory cuffs included the expected CD3^+^ T cells and CD45^+^ macrophages (Supplementary Fig. 3a), and that astrocytes in such inflamed areas were activated, with shorter and thicker processes (Fig. 7b). The astrocytes also showed prominent expression of the glycolytic enzyme PFK2, specifically at the onset of neurological deficits compared with adjuvant-only controls (Fig. 7a,b): such labelling was absent in asymptomatic mice (Fig. 7c), during remission and relapse, and in naïve control mice.

## Discussion

These data show that changes in mitochondrial and neurological function occur in parallel in EAE, suggesting a causal relationship. It is reasonable to suppose that the mitochondrial dysfunction is primary, and this accords with the presence of some mitochondrial deficits in advance of the appearance of neurological signs but worsening significantly at the onset of neurological signs. The changes in mitochondrial function were accompanied by changes in their density, morphology and transport, all of which were spatially related to the presence of inflammatory cells in inflamed lesions. The biochemical activity of mitochondrial complex I was also selectively compromised in spinal tissue at the onset of disease expression. The mitochondrial deficits described above were accompanied by an increase in astrocytic PFK2 expression, consistent with a shift from oxidative to glycolytic ATP production in these cells. Mitochondrial function subsequently improved during remission, and deteriorated again during relapse. The worsening of mitochondrial function on the first day of functional impairment was accompanied by depolarisation of the axons, and the expected conduction failure can explain the close correlation between the mitochondrial and neurological deficits. Indeed, demyelination, which is commonly used to explain the neurological deficits in EAE and MS, was absent at the onset of disease expression in this study, in common with other reports^16^,^17^ so the severe mitochondrial deficits provide the most convincing explanation for the neurological deficits.

The correlation between the exacerbation of mitochondrial dysfunction, axonal depolarisation and the onset and worsening of neurological deficit suggests that a threshold was breached where ATP supply was no longer sufficient to match metabolic demand, and this interpretation fits well with the improvement in mitochondrial function as the animals moved into remission. Indeed, it was striking that mitochondrial function during remission improved to resemble the level observed in asymptomatic animals before the onset of symptoms. During remission, there were more axons that had a normal membrane potential, suggesting that the remission from neurological deficit relied on the restoration of function to a proportion of axons. The severity of mitochondrial and axonal depolarisation therefore correlates quite precisely with the loss of neurological function during relapse, although the traditional explanation would attribute the neurological deficit to demyelination. Demyelination is certainly a potent cause of axonal conduction block^2^,^18^ despite the fact that conduction can also be restored to at least some demyelinated axons even before remyelination^19^,^20^,^21^. It is therefore likely that demyelination will contribute to neurological deficits in disorders where it is abundant, as in MS and in some other types of EAE.

It is likely that the prominent mitochondrial dysfunction that we observe during relapse also contributes to the expression of neurological deficits at this time, although here the balance between mitochondrial dysfunction, demyelination and degeneration is more ambiguous.

Decrease in complex I activity on the first day of loss of neurological function provides a mechanism to explain the decrease in mitochondrial membrane potential reported by decreased TMRM fluorescence. Complex I is the primary gateway for electrons to enter the respiratory chain and a decrease in its activity is expected to decrease mitochondrial membrane potential (for review see ref. 22). Studies in EAE have shown the presence of nitrated proteins on complex I as early as three days post immunisation^23^ indicating evidence of mitochondrial dysfunction.

The cause of the mitochondrial dysfunction remains unclear, although roles for hypoxia, superoxide and nitric oxide are implicated by the current and other studies. Here we have demonstrated real-time and histological evidence for the excessive production of nitric oxide by microglia/macrophages, and nitric oxide is a potent cause of reversible conduction block, particularly in demyelinated axons^7^,^8^. A role for superoxide has been reported in EAE^24^,^25^ and if present with nitric oxide these free radical species form peroxynitrite. Nitric oxide, superoxide and peroxynitrite can compromise mitochondrial function through the inhibition of mitochondrial complexes I to V, aconitase, manganese superoxide dismutase, and creatine kinase^26^; reviewed by this Brown paper^27^, by causing DNA damage and lipid peroxidation, and by increasing mitochondrial proton (and other ion) permeability, probably by lipid peroxidation or thiol cross-linking^28^. Nitric oxide, superoxide and peroxynitrite have also been implicated as responsible for the mitochondrial dysfunction apparent as early as three days after immunisation in excised tissue^23^, and we have confirmed the mitochondrial dysfunction at early days *in vivo* (data not shown), a time when inflammatory cells are very few in number, if present. The importance of nitric oxide and superoxide is supported also by the improvement in neurological deficit in response to inhibition of iNOS by 1400W, or MitoQ, a mitochondrially targeted antioxidant, which we have observed in another model of EAE^29^. A contribution by hypoxia is implicated by related studies of EAE in the rat^29^, where quite profound hypoxia was demonstrated at the onset of neurological deficits. Such hypoxia would be sufficient to contribute to mitochondrial depolarisation, such as that observed here.

In this study, we have focused on axonal mitochondrial function as axonal mitochondria could be reliably distinguished by their CFP label. However, the changes in membrane potential described in axons also apply to the glial cells because the loss of TMRM fluorescence was almost global in inflamed tissue at the onset of neurological deficits, in which case astrocytes and oligodendrocytes must also have been affected.

It is difficult from our findings to determine the sequence of events between the onset of mitochondrial depolarisation and the arrival of any inflammatory cells within the spinal cord. However, it is noticeable that the mitochondrial dysfunction in EAE started before the arrival of many inflammatory cells within the CNS, and initially the dysfunction was not spatially correlated with the locations of inflammatory cells. In contrast, over a few days, a close spatial correlation developed between the location of dysfunctional mitochondria and the accumulation of inflammatory cells. When viewed in clearly inflamed tissue, this correlation has led to the suggestion that the inflammatory cells provoke mitochondrial damage, and indeed, there is a general belief that in both EAE and MS, axonal damage occurs after, and due to, the invading inflammatory cells^1^. However, the current observations suggest that the mitochondrial dysfunction may be primary. The functional mitochondrial deficit that we describe would be undetectable, and so overlooked, by traditional histological methods.

As well as changes in mitochondrial membrane potential during the course of EAE, we also found significant changes in mitochondrial length and mass. The reduction in the mean length of mitochondria in EAE is attributable to fragmentation, because an increase in the number of shorter (and depolarised) mitochondria occurred in conjunction with a decrease in the number of longer mitochondria. The changes in axonal mitochondrial mass (area occupied by mitochondria) were striking, with a small reduction in asymptomatic animals followed by an increase in abundance at the onset of disease expression, an increase that persisted during remission. These changes occurred over only a few days in EAE. The reductions in mitochondrial abundance can be achieved by mitophagy within the axon^30^,^31^ perhaps protecting the neuronal cell body from the damaged mitochondria. An increase in mitochondrial mass has also been reported in MS lesions^32^ and such increases have been interpreted as adaptations to meet an increased energy demand^33^, but the current data suggest that many axonal mitochondria are dysfunctional.

We found that the number of mitochondria trafficking in dorsal column axons was reduced at the onset of neurological deficits, although the axons appeared structurally normal: the reduction in trafficking persisted during remission. The reduction can partly be explained by the large number of depolarized mitochondria, because we have found that only polarized mitochondria move. Mitochondrial trafficking is energetically expensive, and so the reduction in trafficking fits well with our interpretation that the axons have an insufficient supply of energy to maintain their membrane potential and normal function. The reduction in anterograde trafficking would be expected to result in a gradual depletion of mitochondria in the distal parts of axons, as previously observed^34^. This depletion would likely impair the energy production required for effective synaptic function, contributing to the exacerbation of neurological deficits observed. Indeed, a distal energy deficit will also impair synaptic integrity^35^ as observed in EAE^36^ and MS^37^.

The *in vivo* and histological observations reveal that astrocytes are both structurally and functionally damaged in areas of inflammation, especially in association with perivascular cuffs, and similar structural findings have been reported in MS and EAE^38^,^39^. The early increase in astrocytic expression of the enzyme PFK2, a key regulator of glycolysis under several pathophysiological conditions^40^,^41^ is consistent with an upregulation of glycolysis in response to mitochondrial dysfunction^41^. Our data suggest that upregulation of astrocytic glycolysis is only present at very early stages of the disease, which might suggest an attempt to compensate for the decrease in axonal mitochondrial ATP production.

In summary, the current findings focus attention on mitochondrial dysfunction and energy insufficiency as a major cause of neurological deficits in neuroinflammatory disease, providing an explanation for the link between inflammation and major functional deficits in the absence of demyelination. Nitric oxide is implicated by several observations, but tissue hypoxia^29^ may also play an important role. Such metabolic disturbances are not readily detected by conventional histological methods and so they have previously remained largely hidden from view.

## Methods

### Animals

Mice were housed in groups of up to five per cage and had access to food and water *ad libitum*. They were kept at 21 ± 2 °C, on a 12-hour light/dark cycle. EAE was induced in adult female mice, 8–12 weeks-old, transgenic for expression of cyan fluorescent protein (CFP) in axonal mitochondria driven by a neuronal promoter (transgene: Tg (Thy1-CFP/COX8A) S2Lic). The CFP is only expressed in neurons allowing axonal mitochondria to be distinguished from glial and other mitochondria: CFP is expressed selectively in 40–60% of axons^42^.

In total, 105 mice were used in the study, of which 83 were included in the following different analyses. The numbers of animals included in data analysis for *in vivo* imaging experiments were four naïve mice, four asymptomatic mice, three adjuvant-only control mice, five mice on the first day neurological deficits, three mice in remission, and three mice in the chronic relapse stage. For *in vivo* trafficking experiments, four naïve mice were used, four asymptomatic mice, three adjuvant-only control mice, five mice on the first day of neurological deficit, three mice in remission, and in three mice in relapse. For the immunohistological studies, six naive mice were used, six adjuvant-only control mice, six mice on the first day of neurological deficits, three in remission, and six in relapse. For the assay of the activity of the mitochondrial electron transport chain enzymes, six mice were used as adjuvant treated preparations, and six on the first day of neurological deficits.

All experiments were performed in accordance with the UK Home Office Animals (Scientific Procedures) Act (1986) and approved by the local ethics committee (University College London). The experiments were conducted according to the ARRIVE guidelines. All data analysis was carried out in such way that the operator was blinded to the experimental group that was being analysed to minimise bias.

### Experimental autoimmune encephalomyelitis

Mice were immunized with 200 μg of MOG_35–55_ in 200 μl of Incomplete Freund’s adjuvant (IFA) (Sigma, UK) supplemented with 10 mg/ml mycobacterium or saline and adjuvant control by subcutaneous injection, augmented with pertussis toxin, as follows. Day 0: Mice were anaesthetized with isoflurane (2% in room air) and 2 × 100 μl subcutaneous injections of emulsion were made into the sacral haunches 5–10 mm rostral to the base of the tail. Under the same anaesthesia, pertussis toxin (100 μl of 30 ng/ml Bordetella pertussis (Calbiochem, Nottingham, UK)) in sterile saline was injected intraperitoneally (i.p.). Day 1 or 2: A second, similar i.p. injection of pertussis toxin was made. Day 7: Mice were re-immunized with MOG_35–55_/CFA emulsion by injection ∼1 cm rostral to the initial site.

Mice were monitored daily to assess the magnitude of any neurological deficit, and to detect weight gain/loss. The severity of the neurological deficit was scored daily using an eight point grading scale. The signs of EAE appeared approximately 9–14 days after completion of the immunization protocol.

### *In vivo* confocal imaging

Mice were anaesthetized (1.5–2% isoflurane in air) for examination by *in vivo* confocal microscopy on the first or second days disease onset, during remission of neurological deficit, and during relapse (6–8 week p.i.), together with time-matched adjuvant-only controls, and naïve mice. The spinal cord was exposed by removal of the T13-L2 laminae and the animal transferred to a holder in which the vertebral column was rigidly held to minimize movement due to breathing. Mitochondrial membrane potential, a measure of mitochondrial function, was revealed using the cationic, potentiometric dye, tetramethylrhodamine methyl ester (TMRM, Invitrogen, UK) which partitions into polarised mitochondria as a function of membrane potential. TMRM (1 μM solution in sterile saline; pH = 7; Invitrogen, UK) was applied to the exposed spinal cord for 30 minutes using soaked cotton pellets (Coltène/Whaledent Ltd, West Sussex, UK), left in place and hydrated with dye solution immediately after the dura was removed. When required, additional dyes were added to the labelling solution: for example the presence of nitric oxide was detected by application of 4-amino-5-methylamino-2, 7-difluorofluorescein-diacetate (DAF-FM; 1 μM in saline; Invitrogen, UK), a non-fluorescent dye that forms a fluorescent benzotriazole when bound to nitric oxide. Mitotracker Green was used as an additional mitochondrial label that unlike TMRM is membrane potential dependent but not membrane potential sensitive (i.e. unlike TMRM, Mitotracker Green does not leave depolarised mitochondria) (1 μM solution in sterile saline; pH = 7; Invitrogen, UK). The astrocyte specific dye sulforhodamine 101 acid chloride (SR 101) (2 μM solution in sterile saline; pH = 7, Sigma Aldrich, UK) was used. After washing, the mouse was transferred to the customised stage of a confocal microscope (LSM Pascal 5.0, Zeiss, Germany) and the spinal cord imaged using commercial software (LSM Pascal 5.0; Zeiss, Germany) and Zeiss Apochromat Plan x10 and x63 (oil; NA 1.4) warmed (37 °C) objectives. Time-lapse and/or still images were obtained from all exposed areas of the spinal cord. The rectal temperature was maintained at 36–37 °C using a homeothermic heating underblanket (FHC, Maine, and USA).

### Tissue collection and cryosectioning

Following each imaging session the imaged portion of the spinal cord was removed at the level of the lumbar enlargement and post-fixed in 4% glutaraldehyde in 0.1 M phosphate buffer, and prepared for high resolution light microscopy (See supplementary methods).

In addition spinal cord tissue was also taken from mice unimaged for all groups (n = 6 navies, n = 6 adjuvant only treated, n = 6 1st day neurological deficits, n = 3 remission and n = 6 mice in relapse) and used for immunohistochemical analysis. In brief, mice were anaesthetized with 2% isoflurane in air then transcardially perfused with cold PBS followed by 4% paraformaldehyde. The spinal cord was then removed kept in 4% paraformaldehyde overnight then transferred to 30% sucrose supplemented with sodium azide until processed for immunohistochemistry.

Transverse and longitudinal sections of the spinal cords were embedded in OCT (Jung tissue freezing medium) and cut as 10 μm thick, free floating, sections using a cryostat (Leica CM1950) at −20 °C. Sections were maintained in PBS with 0.1% sodium azide until used for immunohistochemistry.

### Image analysis

Mitochondrial number, length, membrane potential and abundance were assessed using Image J (version 1.43; NIH, Bethesda, MD, USA, rsb.info.nih.gov/ij/) (See supplementary methods 1). The mitochondria routinely appeared to be ~0.5 μm in width, and only their length varied: the length was therefore used as a measure of mitochondrial size throughout.

To assess the axonal ‘mitochondrial power’ in the complex spinal tissue we have sought to measure the abundance of polarised axonal mitochondria and their degree of polarisation, to create an ‘index of axonal mitochondrial capacity’. To achieve this we multiplied the combined length of the polarised axonal mitochondria (i.e. the area under the frequency distribution curve of TMRM^+^ mitochondrial length per mouse), by their mean fluorescent intensity (expressed by the mean coefficient of variance of TMRM pixel intensities; see supplementary methods 1).

At the end of an imaging session, upon death of the animal, the TMRM fluorescence dissipated and the fluorescence intensity never increased, showing that TMRM was not quenching.

To assess the axonal membrane potential between different groups the mean intensity of the TMRM fluorescence in the axoplasm, avoiding the inclusion of mitochondria, was measured and expressed as a ratio of the mean intensity of the (non-fluorescent) blood in nearby vessels. Blood vessels were chosen due to lack of identifiable extracellular space in the dorsal column, which ideally would provide a better measure. The number of axons and animals analyzed in the different groups was respectively n = 83 axons in four naïve mice; n = 140 axons in three adjuvant treated mice, n = 90 axons in four asymptomatic mice, n = 137 axons in five mice on the first day of neurological deficits, n = 63 axons in three mice during remission and n = 122 in three mice in relapse.

### Mitochondrial Trafficking

The number, direction and velocity of moving mitochondria in time-lapse movies recorded over 50 frames (i.e. 98.5 s) were analysed using Image J. (See supplementary methods).

### Statistical analysis

The data were tested for normality of distribution using the D’Agostino and Pearson normality test. Parametric data are presented as mean ± S.E.M. unless indicated otherwise. Comparison between axonal mitochondrial TMRM intensity (measure of function), mitochondrial length, mitochondrial abundance, number of motile mitochondria and average speed of motile mitochondria per axon were all made by two-way ANOVA using GraphPad Prism software (GraphPad, San Diego, CA) accompanied with Bonferroni post-hoc analysis. All comparisons were done within and between groups. p < 0.05 was considered significant (*p < 0.05; **p < 0.01; ***p < 0.001).

## Additional Information

**How to cite this article**: Sadeghian, M. *et al.* Mitochondrial dysfunction is an important cause of neurological deficits in an inflammatory model of multiple sclerosis. *Sci. Rep.* **6**, 33249; doi: 10.1038/srep33249 (2016).

## Supplementary Material

Supplementary Video 1

Supplementary Video 2

Supplementary Video 3

Supplementary Information
