# Supplementary material for: Mitochondrial dysfunction is an important cause of neurological deficits in an inflammatory model of multiple sclerosis
Source: Sci Rep. 2016 Sep 14;6:33249. doi: 10.1038/srep33249 (PMC5021937; doi:10.1038/srep33249)
Supplement: Supplementary Information [file srep33249-s4.pdf]

Mitochondrial dysfunction is an important cause of neurological deficits in an inflammatory model of multiple sclerosis.

Mona Sadeghian[1], Vincenzo Mastrolia[1] Ali Rezaei Haddad[1], Angelina Mosley[1], Gizem Mullali[1], Dimitra Schiza[1], Marija Sajic[1], Iain Hargreaves[2], Simon Heales[3], Michael R Duchen[4], Kenneth J Smith[1]

1 Department of Neuroinflammation, Queen Square Multiple Sclerosis Centre, UCL Institute of Neurology.

2 Neurometabolic Unit, National Hospital for Neurology and Neurosurgery, London, UK

3 Chemical Pathology, Great Ormond Street Children's Hospital, London, UK

4 Cell and Developmental Biology, University College London, Gower Street, London.

Correspondence to: Dr Mona Sadeghian

Email: m.sadeghian@ucl.ac.uk

Supplementary methods

1-Image analysis

To determine the size and number of mitochondria for each image (acquired as the cyan channel for CFP^+ mice or the red channel for TMRM) the background intensity was subtracted and the resulting image was transformed to a binary image. Using the Analyze Particle plug-in from image J, and setting appropriate ranges of size and circularity to remove false positive particles, all mitochondria present in the parenchymal areas of the binary images were distinguished and their length determined. The analysis included 400 axonal mitochondria from n= 27 axons and n=35 axons in each of four naïve and four asymptomatic mice respectively, 320 axonal mitochondria from n= 35 axons in each of three adjuvant control mice, 450 axonal mitochondria from n= 95 axons in each of five mice examined on the first day of neurological deficit, and 360 axonal mitochondria from n= 66 axons and 350 mitochondria from n=65 axons examined in each of three mice in remission, and in three mice in relapse (6-7 weeks after immunisation) respectively.

Mitochondrial function was assessed by the intensity of their fluorescence for TMRM. If axonal mitochondria exhibited no detectable TMRM fluorescence ($CFP^+/TMRM^-$) they were regarded as totally depolarised, but if they exhibited visible TMRM fluorescence ($CFP^+/TMRM^+$) they were regarded as being polarised, even if partially depolarised. The analysis was therefore conducted in two steps. First, the simple presence or absence of any distinguishable TMRM fluorescence in axonal mitochondria in the CFP^+ mice was determined using a colocalization plugin in MacBiophotonics Image J version. The percentage of $CFP^+/TMRM^-$ mitochondria was used as one measure of the magnitude of loss of mitochondrial function. Second, in $CFP^+/TMRM^+$ mitochondria the intensity of fluoresence was expressed as the coefficient of variance of TMRM pixel intensities of mitochondria against axonal TMRM itensities (standard deviation of the mean intensity divided by the mean

intensity)[1], providing a representation of the spatial heterogeneity of intracellular dye distribution between the axoplasm and mitochondrial compartments. This measure indicates the ability of mitochondria to concentrate the TMRM, i.e. their membrane potential, which is independent of mitochondrial mass, number or density.

2-Mitochodrial trafficking

Segments 100μm long of myelinated axons 2-5μm in diameter were first straightened using the Straighten plug-in tool (http://rsbweb.nih.gov/ij/plugins/straighten.html) and the images were then stabilized to correct for breathing artifacts using the StackReg plugin (http://bigwww.epfl.ch/thevenaz/stackreg/). Straightened axons were processed using the Difference Tracker plug-in (http://www.bioinformatics.bbsrc.ac.uk/projects/difference_tracker/) for ImageJ as previously described [2, 3]. Mitochondrial structures in which at least 6 pixels were moving over at least 4 consecutive frames (i.e. 7.9s) were considered as moving. The lower cut off was determined by examination of the size of the smallest moving mitochondria (CFP and TMRM positive particles). The number of moving mitochondria, and their direction and velocity were directly derived from the results table generated by the Mass Particle Tracker part of the Difference Tracker plug-in. The time-lapse videos were analysed from four different groups of mice: naïve (n=89 axons from three animals); first day of expression of neurological deficit (n=76 axons from three animals); adjuvant control (time matched with first day mice; n=35 axons from two animals); and remission (n=67 from 3 mice). The time-lapse videos and the images from the four different groups were analyzed blindly.

3- Activity of enzymes in the mitochondrial respiratory chain

To corroborate our findings on decreased TMRM signal as a read out for mitochondrial dysfunction from the *in vivo* imaging experiments we measured the activity of mitochondrial respiratory chain enzymes on the first day of neurological defects. A separate cohort of mice

(n= 6 EAE and n=6 adjuvant only controls) were immunised to induce EAE (see Methods). On the first day of neurological signs the animals were perfused with PBS and the spinal cord was freshly dissected and snap frozen in liquid nitrogen. Tissue homogenates were prepared as described by Heales et al (1996). Tissue samples (50-100 mg) were homogenised 1:9 (w/v) in media: 320 mmol/L, 1 mmol/L ethylenediamine tetra acetic acid dipotassium salt, 10mmol/L Trizma-base.

4-Enzymatic assays

Prior to analysis homogenates were subjected to three cycles of freezing and thawing to lyse membranes. Enzyme activities were assessed at 30°C using a Uvikon 940 spectrophotometer (Kontron Instruments Ltd, Watford, UK). Complex I activity was measured according to the method of Ragan *et al* (1988), whereas complex II-III and complex IV activities were measured according to the methods of King (1967) and Wharton and Tzagoloff (1967) respectively. Citrate synthase (CS; EC 1.1.1.27) activity was determined by the method of [5]. Enzyme activities are expressed as a ratio to citrate synthase (mitochondrial marker enzyme) to compensate for mitochondrial enrichment in the cell samples [6]. The difference between groups for each enzyme activity assay was measured by two tailed unpaired *t* test, ** = $p < 0.01$.

5- Immunohistochemical and histochemical labelling

Luxol Fast Blue/Periodic Acid Schiff

Sections were dehydrated in ascending concentrations of ethanol (5min each) starting at 70% followed by 90% then 100%. Sections were immersed in 0.1% LFB in 95% alcohol at 50°C overnight and washed under running tap water followed by incubation in saturated lithium carbonate (20-60 sec). After washing under running tap water sections were oxidized in 1% periodic acid (20 min) followed by washing under running tap water. Sections were placed in cold fresh Schiff's reagent (10 min) and washed well under running tap water (10

min), and then immersed in Harris' haematoxylin (5 min) and washed with tap water (5 min) before incubating in 1% acid alcohol for (30 sec) followed by a wash with tap water. Dehydration and defatting processes were carried out by incubating the sections through ascending concentrations of ethanol to xylene (70%, 90%, 100% ethanol, xylene I, xylene II), then sections were mounted using DPX mounting medium.

High resolution microscopy

Following each imaging session the imaged portion of the spinal cord was removed at the level of the lumbar enlargement and post-fixed in 4% glutaraldehyde in 0.1 M phosphate buffer, and prepared for high resolution light microscopy by embedding into TAAB resin (TAAB Laboratories) using a standard protocol as previously described[7]. Following resin embedding, semi-thin transverse sections (700 nm) were cut, collected onto slides, and stained with toluidine blue. These sections were examined at high magnification.

Fluorescence immunohistochemistry

Longitudinal and transverse sections of the spinal cord tissue were washed in 0.1M PBS (5 min) followed by antigen retrieval using heat treatment in citric acid (20 min). Sections were placed in cold methanol (30 min) and washed in PBS Triton-X (PBSTX) (0.1% Triton-X-100) 3x 5min before incubation in appropriate sera to block non-specific binding. Sections were incubated with the primary antibody at the appropriate dilution in PBS triton X (PBS-TX) overnight (see below). The following day the sections were washed with PBS-TX (3x 5min) and then the appropriate secondary antibody with fluorescent conjugate was applied in normal serum and PBS-TX (1 hour), in the dark. Slides were washed in PBS (3x 5min) and finally rinsed with distilled water and left to air dry in the dark.

The following single and double immunofluorescence labellings were performed: 3-nitrotyrosine (1:200), mouse monoclonal (Millipore, UK) was used to detect the presence of nitrated residues; SMI-32 (1:1000), mouse monoclonal (Abcam, UK) was used to detect

hypophosphorylated neurofilaments, a marker for axonal damage; iNOS (1:200) rabbit polyclonal (Abcam, UK) was used to detect the presence of the inducible form of nitric oxide synthase, in combination with CD45 (1:2000), rat monoclonal (Abcam, UK) that detects the infiltration of hematopoietic cells in the tissue. OX-6 (1:250) mouse monoclonal (Abcam, UK) that detects MHC II expressing activated microglia. Iba1 (1:500) goat polyclonal (Abcam, UK) general microglial marker. GFAP (1:1000), rabbit poly clonal (Abcam, UK) was used to detect the glial fibrillary acidic protein expressed by astrocytes, in combination with PFK-2 (1:250), goat polyclonal IgG (Santa-Cruz, USA). PFK-2 was used to detect the presence of the enzyme phosphofructokinase-2 in astrocytes, an enzyme involved in the regulation of glycolysis and gluconeogenesis.

Supplementary Figures

Supplementary Fig 1 Sadeghian

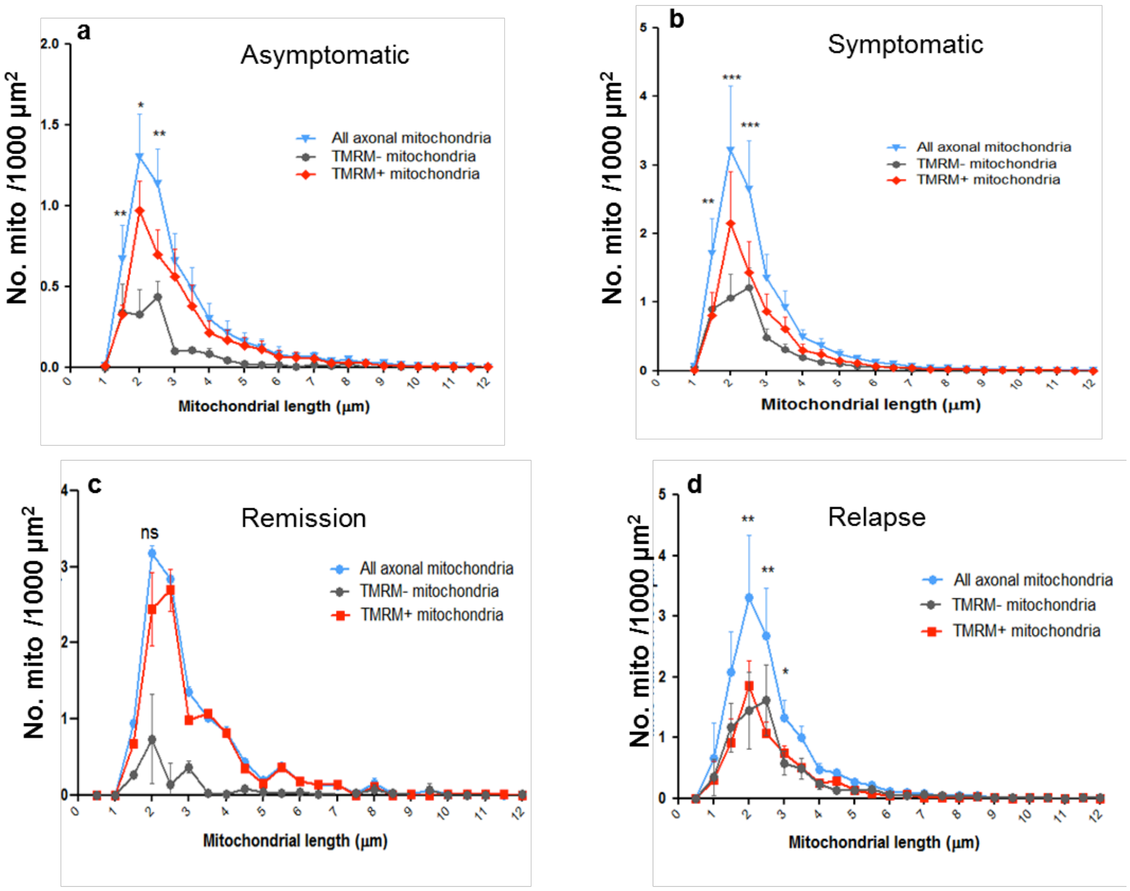

Supplementary Figure 2 Sadeghian

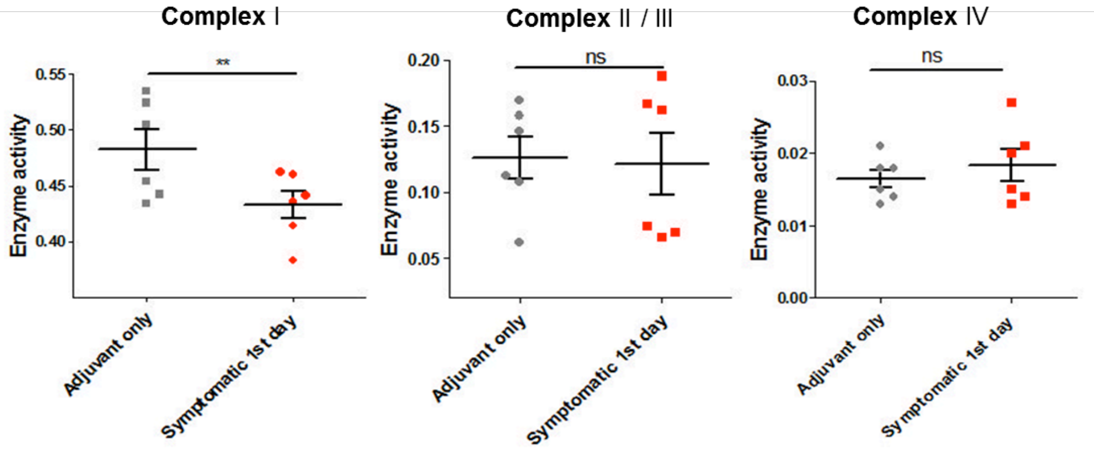

Supplementary Fig 3 Sadeghian

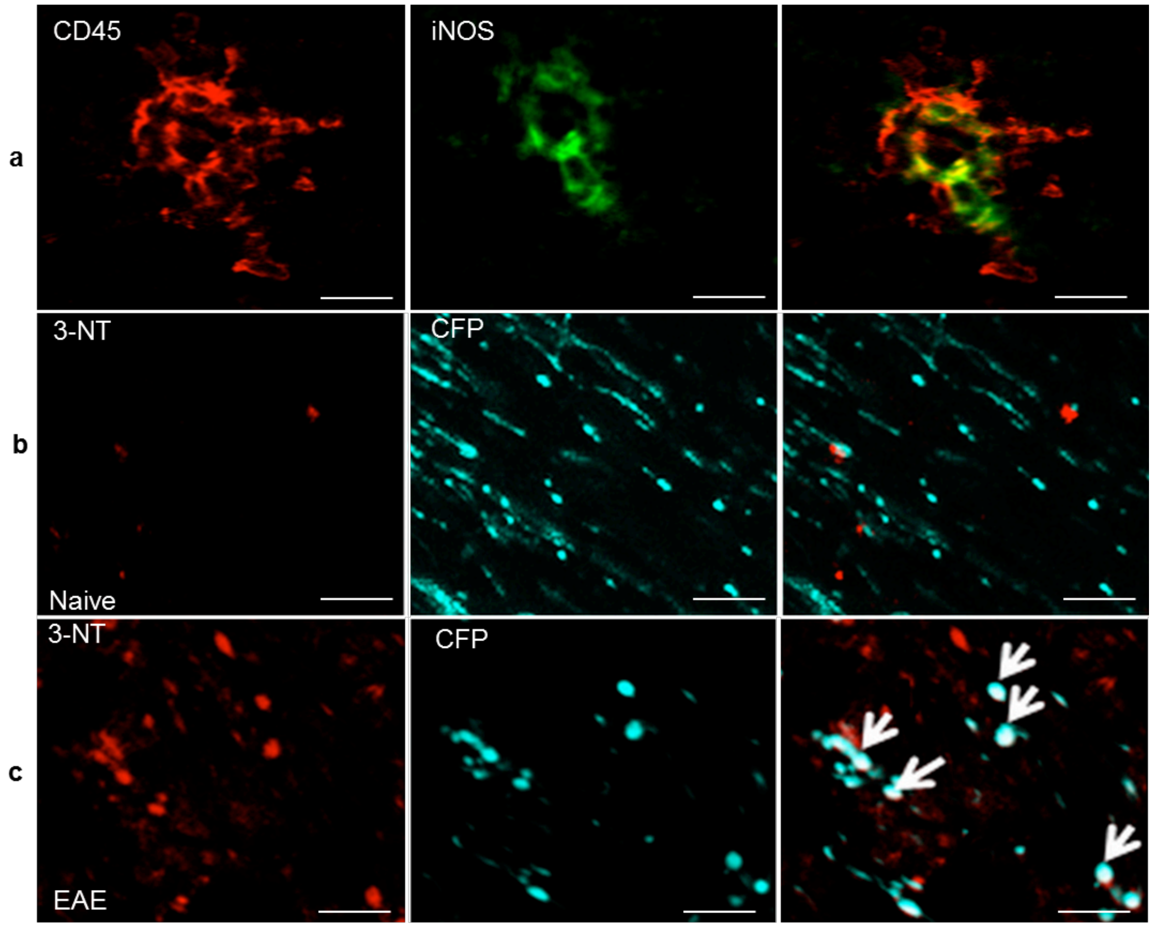

Supplementary Fig 4 Sadeghian

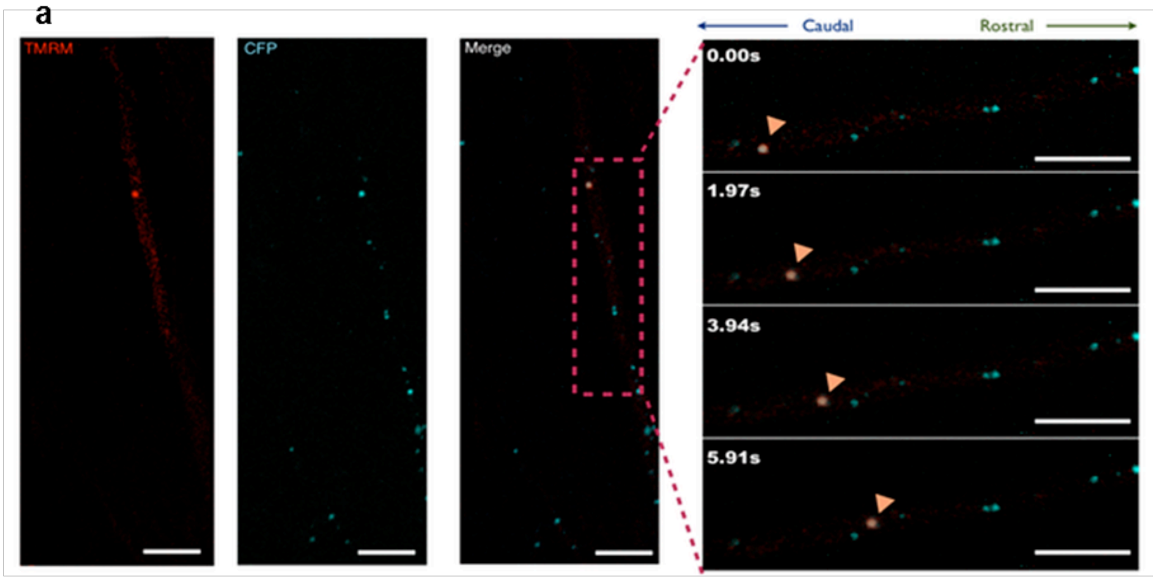

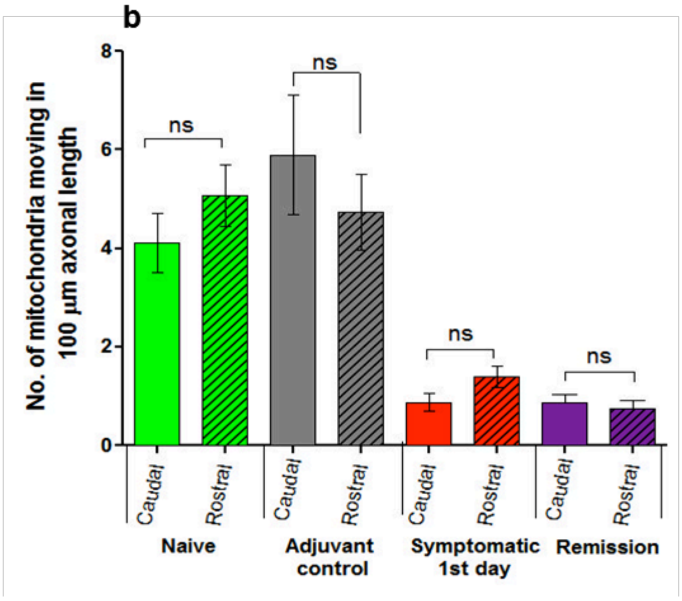

Supplementary Figure 5 Sadeghian

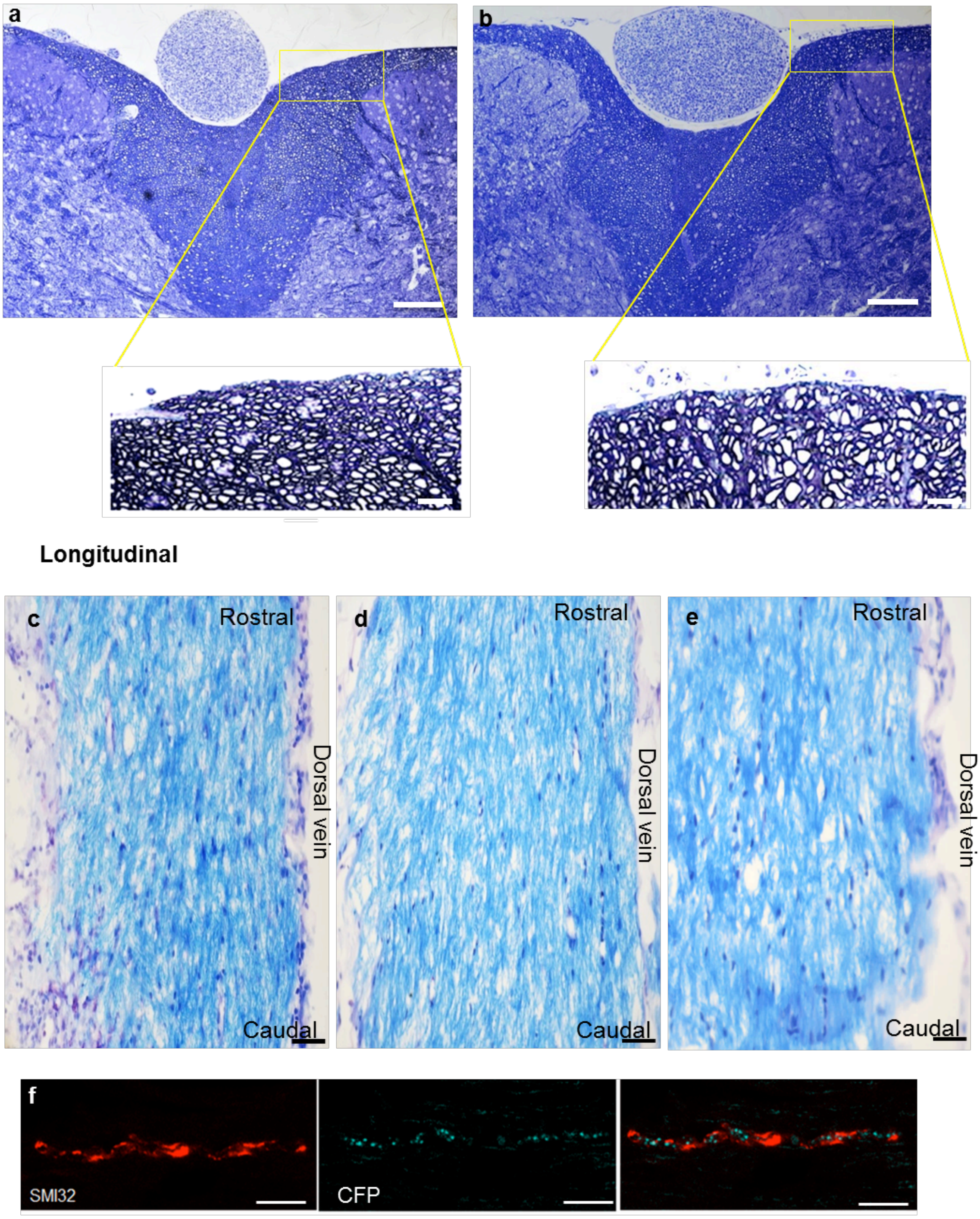

Supplementary Figure legends

Supplementary Figure 1: (**a-d**). Graphs showing the frequency distribution of mitochondria with any detectable TMRM fluorescence (red), and of totally depolarised mitochondria (grey), versus their length, at the indicated stages of EAE (the normal distribution is shown in figure 4). Whereas virtually all mitochondria were brightly fluorescent in naïve and adjuvant-only control animals (figure 4), many were completely or almost completely depolarised (showing CFP but no detectable TMRM fluorescence) in animals at all studied stages of EAE. (**a**) In asymptomatic animals, 36% of small axonal mitochondria (1.5-2.5 µm in length) lacked any detectable TMRM fluorescence), comprising 28% of the total axonal mitochondrial mass (area under the curves). (**b**) On the first day of neurological signs the proportion of depolarised axonal mitochondria increased to 54% of the small mitochondria (1.5-2.5 µm in length), comprising 40% of the total axonal mitochondrial mass (area under the curves). (**c**) In mice during remission, 19% of the total mass of axonal mitochondria was entirely depolarised (area under the curves). (**d**) During relapse the proportion of depolarised axonal mitochondria deteriorated and approximately half were TMRM$^-$ (area under the curves). Comparisons in each group were made by two-way ANOVA using GraphPad Prism software (GraphPad, San Diego, CA) accompanied with Bonferroni post-hoc analysis. .* = $p < 0.05$, ** = $p < 0.01$, *** = $p < 0.001$.

Supplementary Figure 2: Graphs showing mitochondrial respiratory chain enzyme activity. (**a**) Significant reduction in mitochondrial complex I activity on the first day of neurological deficits compared with adjuvant only control mice. (**b, c**) Complex II/III and IV activities were unchanged between the two groups. Each data point represents the enzyme activity per spinal cord per mouse.

Supplementary Figure 3: Immunohistochemical labelling for the expression of iNOS and 3-NT by macrophages and axonal mitochondria respectively in the mouse spinal cord. (**a**) On the first day of expression of neurological deficit due to EAE, iNOS labelling (green) colocalises with $CD45^+$ (red) macrophages, appearing yellow in the merged images (right). (**b**) Naïve animals lack immunohistochemical labelling for 3-NT in CFP^+ axonal mitochondria in longitudinal sections from the spinal cord, but (**c**) on the first day of neurological deficits such mitochondria become positive for 3-NT labelling (white arrows). The CFP^+, 3-NT^+ axonal mitochondria appear morphologically fragmented compared with naïve animals. (Scale bar 10μm).

Supplementary Figure 4: (**a**) Confocal images of the spinal cord of an anaesthetised mouse showing movement of a polarized small mitochondrion, and lack of movement of several depolarized mitochondrial fragments, in an axon on the first day of neurological deficits. The uppermost CFP^+ mitochondrion is positive for TMRM, but the several other CFP^+ mitochondria are depolarized ($TMRM^-$). The fourth panel illustrates a time-lapse sequence of mitochondrial trafficking in the indicated area: the arrowhead points to the functional CFP^+/$TMRM^+$ mobile mitochondrion, which moves over time, whereas the several depolarized mitochondria remain immobile. Scale bar 10μm. (**b**) Graph showing that the direction of mitochondrial movement along dorsal column axons was relatively balanced in naïve animals and in animals at different stages of EAE, although many fewer were motile in EAE. Data were collected from n=89 axons from three naïve animals; n=35 axons from two adjuvant control mice that were time matched with n=76 axons from three mice examined on the first day of expression of neurological deficit; and from n=67 axons from three mice in remission. Comparisons were made by two-way ANOVA using GraphPad Prism software

(GraphPad, San Diego, CA) accompanied with Bonferroni post-hoc analysis. All comparisons were done within and between groups.

Supplementary Figure 5: (**a, b**) Histological evidence showing the lack of demyelination and axonal degeneration on the first day of neurological deficits. (**a**) Photomicrographs showing the lumbar spinal cord post imaging in high resolution, transverse resin sections (700 nm thick) stained with toluidine blue, with inserts at higher magnification, from a naïve (**a**) animal and one on the first day of neurological deficits (**b**). Apart from the presence of some inflammatory cells in B, the appearance of the two sections is similar, and neither shows any demyelinated or degenerated axons. (**c-e**) Luxol fast blue (LFB) labelling of longitudinal spinal cord sections reveals no significant demyelination in EAE (**c**) compared with naïve and adjuvant treated mice (**d**,**e**, respectively): myelin appears blue and cell bodies, including the infiltrating immune cells, appear magenta. (**f**) Longitudinal section through the dorsal columns showing an $SMI32^+$ axon on the first day of neurological deficit containing many short mitochondria. (Scale bar 10μm).

Supplementary videos

Video 1: Time-lapse video of macrophages labelled with TMRM (red) and DAF-FM diacetate (green) in the dorsal column imaged *in vivo*. Presence of green fluorescence (DAF-FM diacetate) is indicative of nitric oxide production.

Video 2: (**a**) Time-lapse video of trafficking mitochondria in the dorsal column axons of a naïve mouse. The majority of axonal mitochondria are CFP^+ (blue mitochondria), the red signal is TMRM labelling of functional mitochondria. A small population of axonal mitochondria traffic along the dorsal column axons, with movement in both directions, but the majority of mitochondria are stationary. The mean number of motile axonal mitochondria in control dorsal column axons is 8.58 ± 1.11 per 100μm axonal length in naïve animals.

(**b**) Time-lapse video of trafficking mitochondria of dorsal column axons at the onset of neurological deficits. Mitochondrial trafficking was significantly reduced such as the mean number of motile axonal mitochondria in the dorsal column axons was only 2.04 ± 0.34 per 100μm axonal length on the first day of neurological deficits in EAE ($p<0.001$ compared with control mice). The majority of CFP^+ axonal mitochondria have either no, or a very low level, of TMRM labelling.

Reference List

1. Duchen,M.R., Surin,A., & Jacobson,J. Imaging mitochondrial function in intact cells. *Methods Enzymol.* **361**, 353-389 (2003).

2. Andrews,S., Gilley,J., & Coleman,M.P. Difference Tracker: ImageJ plugins for fully automated analysis of multiple axonal transport parameters. *J. Neurosci. Methods* **193**, 281-287 (2010).

3. Sajic,M. *et al.* Impulse conduction increases mitochondrial transport in adult mammalian peripheral nerves in vivo. *PLoS. Biol.* **11**, e1001754 (2013).

4. Shepherd,D. & Garland,P.B. The kinetic properties of citrate synthase from rat liver mitochondria. *Biochem. J.* **114**, 597-610 (1969).

5. Heales,S.J., Bolanos,J.P., Brand,M.P., Clark,J.B., & Land,J.M. Mitochondrial damage: an important feature in a number of inborn errors of metabolism? *J. Inherit. Metab Dis.* **19**, 140-142 (1996).

6. Redford,E.J., Hall,S.M., & Smith,K.J. Vascular changes and demyelination induced by the intraneural injection of tumour necrosis factor. *Brain* **118 (Pt 4)**, 869-878 (1995).

7. Ragan CI, Wilson MY, Darley-Usman VM, et al (1988) Subfractionation of mitochondria and isolation of proteins of oxidative phosphorylation. In Darley VM, Rickwood D, Wilson MT, eds. *Mitochondria: A Practical Approach*. Oxford : IRL Press, 79-113.

8. King TE (1967) Preparation of succinate cytochrome c reductase and cytochrome b-c1 particle and reconstruction of Succinate cytochrome c reductase. *Methods Enzymol* **10**: 446-51.

9. Wharton DC, Tzagoloff A (1967) Cytochrome oxidase from beef heart mitochondria. *Methods Enzymol* **10**: 245-50.
